# The globular domain of extracellular histones mediates cytotoxicity *via* membrane disruption mechanism

**DOI:** 10.1016/j.jbc.2024.108038

**Published:** 2024-11-28

**Authors:** Yixuan Pan, Mengyuan Peng, Mindan Tong, Yue He, Min Hao, He Lilian Gao, Yimin Lao, Jingdong Xue, Meiyang Liu, Qing Zhong, Xiaoxia Liu, Bing Li

**Affiliations:** 1 Department of Biochemistry and Molecular Cell Biology, Shanghai Key Laboratory for Tumor Microenvironment and Inflammation, Key Laboratory of Cell Differentiation and Apoptosis of Chinese Ministry of Education, Shanghai Jiao Tong University School of Medicine, Shanghai, China; 2 Key Laboratory of Cell Differentiation and Apoptosis of Chinese Ministry of Education, Department of Pathophysiology, Shanghai Jiao Tong University School of Medicine, Shanghai, China

**Keywords:** membrane leakage, cytotoxicity, extracellular histone, histone globular domain, cell death, membrane bilayer, liposome, histone, extracellular matrix

## Abstract

Histones are traditionally recognized for structuring nuclear architecture and regulating gene expression. Recent advances have revealed their roles in inflammation, coagulation, and immune responses, where they act as damage-associated molecular patterns. The mechanisms by which histones induce membrane leakage are not well understood, and certain cells, including endothelial cells and peritoneal macrophages, show resistance to histone-mediated pore formation. We utilized liposome leakage assays to explore the pore-forming capabilities of different histone configurations, including individual histones, tail regions, and globular domains. Our results demonstrate that globular domains primarily drive pore formation. Using cytotoxicity assays, we further demonstrate that the globular domain of extracellular histones is primarily implicated in inducing lytic cell death. This study provides insights into the pathological roles of histones and suggests potential therapeutic targets to mitigate their harmful effects.

Histones have long been recognized primarily for their fundamental roles in structuring nuclear architecture and modulating gene expression (1). However, recent advancements in molecular biology have unveiled a range of non-canonical functions that extend beyond these traditional roles (2). For instance, Attar *et al.* have demonstrated that the histone H3-H4 tetramer functions as a copper reductase enzyme (3). This enzyme actively catalyzes the reduction of copper ions from Cu^2+^ to the more bioavailable Cu^1+^ state (3), revealing a previously unappreciated biochemical capability of these nuclear proteins. Beyond their intracellular roles, histones, along with DNA, have been observed to exert substantial extracellular effects, particularly following their release from cells during cellular death. This release mechanism is not merely a consequence of cellular demise but has been implicated in broader biological processes and pathologies (4). The presence of extracellular histones and DNA is increasingly recognized for its potential implications in inflammation, coagulation, and immune responses, wherein these molecules may act as damage-associated molecular patterns (DAMPs) (2). In the context of cancer, DNA from neutrophil extracellular traps (NETs) is prevalent in liver metastases from breast and colon cancers. This DNA functions as a chemotactic agent that attracts cancer cells, thereby promoting the formation of distant metastases (5). Additionally, while nucleosomes have been found to induce lymphocyte necrosis (6) and neutrophil activation (7), histones themselves do not trigger these responses, suggesting that their functions are not confined to the nucleus but are integral to cellular and systemic homeostasis.

Extracellular histones influence cellular functions through diverse mechanisms that can be categorized primarily into membrane protein-dependent and membrane protein-independent pathways. Membrane protein-dependent pathways typically involve histones binding to specific cell surface receptors or membrane proteins, which initiate signal transduction pathways influencing cellular behavior. Extracellular histones are significant mediators of death in sepsis, contributing to mortality through inflammatory and chemically induced cellular injuries (8). These actions are mediated in part by interactions with Toll-like receptors (TLRs), specifically TLR2 and TLR4, which play critical roles in initiating and propagating inflammatory responses (9). Likewise, extracellular histones from dying renal cells directly interact with TLR2 and TLR4 to activate downstream signaling and aggravate kidney injury (10); Extracellular histones can also inhibit CNS axonal regeneration *via* the transcription factor Y-box-binding protein 1 and Toll-like receptor 2 (11). Additionally, histones interact with receptors for advanced glycation end-products (RAGE), which further amplifies inflammatory and stress responses within cells (4). Moreover, histone extracts from *Plasmodium falciparum*, the causative agent of the most severe form of malaria, including H3 and H4, induced IL-8 production and endothelial permeability (12). In trauma-associated lung injury, extracellular histone toxicity originates from their affinity for phospholipids, which leads to their integration into cell membranes and causes large inward ion currents and calcium influx (13). Histone H4 has been well known as a major component of the antimicrobial action of human sebocytes (14). Lastly, extracellular histones can directly induce autophagy and apoptosis in a dose-dependent manner in cultured human endothelial cells (15).

In contrast, membrane protein-independent mechanisms involve the direct action of histones on the cell or its components without the mediation of specific membrane receptors. Histones can induce coagulation by acting directly on clotting factors, thereby promoting thrombin generation and fibrin formation, which are crucial to causing profound thrombocytopenia (16). Extracellular histones can cause direct cytotoxic effects by compromising the integrity of cellular membranes presumably through electrostatic interactions, leading to cell lysis and death (2). A breakthrough study reports that histone H4 from neutrophil extracellular traps binds to the membranes of smooth muscle cells (SMCs) and causes leakage, lysing the SMCs, which triggers arterial tissue damage, inflammation, and the destabilization of plaques (17). The neutralization of histone H4 by adding APC protease prevents cell death of SMCs and stabilizes atherosclerotic lesions (17). Membrane disruption typically occurs when amphiphilic peptides or proteins, acting as pore-forming agents, interact with bilayers to form oligomeric pores (18). These pores, varying in size up to tens of nanometers, enable the transport of molecules and materials (18). Various intrinsic pore-forming proteins such as apoptosis regulator BAX (BAX), BCL-2 homologous antagonist/killer (BAK), BCL-2-related ovarian killer protein (BOK), Gasdermin family members (GSDMs), and Mixed Lineage Kinase Domain-Like protein (MLKL) create diverse pore architectures in different cell death pathways (19). However, the mechanism by which histones induce membrane leakage remains largely unclear. Importantly, cellular responses suggest some cells, such as endothelial cells and peritoneal macrophages (17), are resistant to histone-mediated pore formation, raising questions about the source of this specificity.

To address these questions, we employed a biochemical strategy, utilizing liposome leakage assays to probe the pore-forming capacities of various histone forms, including individual histones, tail regions, and globular domains. Unexpectedly, our results suggest that the globular domains contribute most significantly to pore-forming activity. Subsequently, we explored the combination of different histone forms using cytotoxicity assays to further elucidate the mechanisms underlying histone-induced cellular damage. Our deeper mechanistic understanding of how histones affect membrane integrity under pathological conditions may inspire new targeted strategies to mitigate their harmful effects.

## Results

### Membrane leakage can be directly induced by individual histones

**Figure 1:**
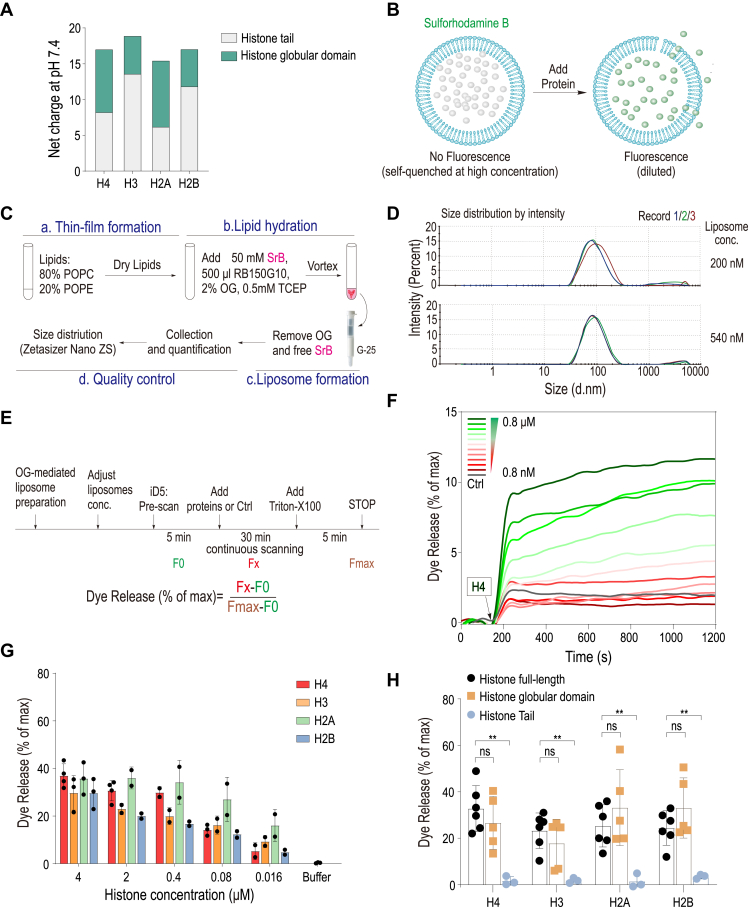
**Liposome Membrane leakage can by directly induced by individual histones.***A*, net electrostatic charge of four canonical histones at pH 7.4, highlighting the charge distribution between the histone tail and globular domain. *B*, diagram illustrating the liposome leakage assay, wherein disruption of the liposome membrane leads to the release of self-quenched sulforhodamine *B*, resulting in a detectable fluorescence signal. *C*, flowchart of the process for detergent-mediated liposome preparation. SrB stands for sulforhodamine *B*. *D*, size distribution profile of the liposomes prepared as described. *E*, flowchart detailing the steps of the liposome leakage assay. Dye efflux is quantified as a percentage of the maximal release triggered by detergent exposure. *F*, rapid leakage from liposomes induced by histone H4, observed over a concentration range from 0.8 nM to 0.8 μM across various time points. *G*, significant leakage from liposomes was observed with all tested histone types. *H*, globular domains of each histone contribute to membrane leakage. A comparative analysis of liposome leakage activity among full-length histones, histone tails, and globular domains, all tested at a concentration of 2 μM. Results are representative of at least three independent experiments.

Among canonical histones, histone H4 has been shown to possess the most potent membrane pore-forming capability, an attribute primarily based on its cationic N-terminal domain which is thought to induce plasma membrane bending and subsequent pore formation (17). However, our calculations of the net charge across the full-length core histones, as well as their respective tail and globular domains, indicate that all histones possess a substantial positive charge, with histone H3 displaying the highest (Figs. 1*A* and S1). This discrepancy suggests that factors other than mere charge may play significant roles in the differential pore-forming abilities of histones. To further investigate the direct effects of histones on membrane permeabilization, we utilized a liposome leakage assay employing a combination of highly purified individual histones (Fig. S1*B*).

In this assay, protein-free liposomes encapsulating sulforhodamine B at self-quenching concentrations were subjected to incubation with various histones. The principle behind this is that protein-induced leakage of the liposomal content leads to dilution of sulforhodamine B, increasing in fluorescence intensity, which serves as a quantitative measure of membrane disruption (Fig. 1*B*). Liposomes were reconstituted using a detergent-based method (20). Dynamic light scattering (DLS) was subsequently employed to verify the size distribution of the liposomes (Fig. 1*C*). Representative DLS spectra from two different liposome concentrations showed their similar size profiles, with diameters predominantly distributed around 100 nm, confirming the consistency of liposome preparation (Fig. 1*D*). These liposomes consisted of a lipid composition of 80% zwitterionic POPC and 20% negatively charged POPE. This common lipid makeup was chosen considering its potential interaction with cationic proteins such as histones. The release of liposomal content was monitored by measuring the increase in sulforhodamine B fluorescence, the extent of which was quantified as a percentage of the total fluorescence observed after complete liposome disruption with Triton X-100 after each assay (Fig. 1*E* *bottom*).

To establish the dynamic range of histone-induced membrane permeabilization, we initially focused on histone H4. Our experiments demonstrated that the addition of H4 led to rapid and dosage-dependent liposome leakage (Fig. 1*F*). Following this observation, we proceeded to quantify the leakage activity of all four core histones by measuring the extent of liposome leakage after the addition of various concentrations of each histone. The results from these assays revealed that all core histones possess the capability to destabilize lipid bilayers, with notable differences in their efficacy (Fig. 1*G*). Specifically, histones H4 and H2A exhibited a higher degree of leakage activity compared to histones H3 and H2B (Fig. 1*G*). This differential activity underscores the variability in membrane interaction across different histone types and suggests that biochemical differences among the histones may influence their membrane-destabilizing potential.

The documented ability of a fusion protein comprising a histone tail and a cell-penetrating peptide derived from HIV TAT to translocate into the nucleus while preserving cell membrane integrity (21) suggests that the histone tail component does not primarily mediate membrane disruption. To further delineate the regions within histones responsible for active pore formation, we conducted comparative analyses using the liposome leakage assay, focusing on full-length histones, their globular domains, and histone tails. Our results surprisingly indicated that the globular domains of all four core histones exhibited membrane permeabilization activity comparable to that observed with full-length histones (Fig. 1*H*). In stark contrast, the histone tails alone did not induce any liposome leakage (Fig. 1*H*). These findings underscore the significance of the globular domains in mediating membrane interactions and suggest that they contain the primary structural elements responsible for the pore-forming capabilities of histones.

### Extracellular histones exhibit cytotoxicity across various cell types

**Figure 2:**
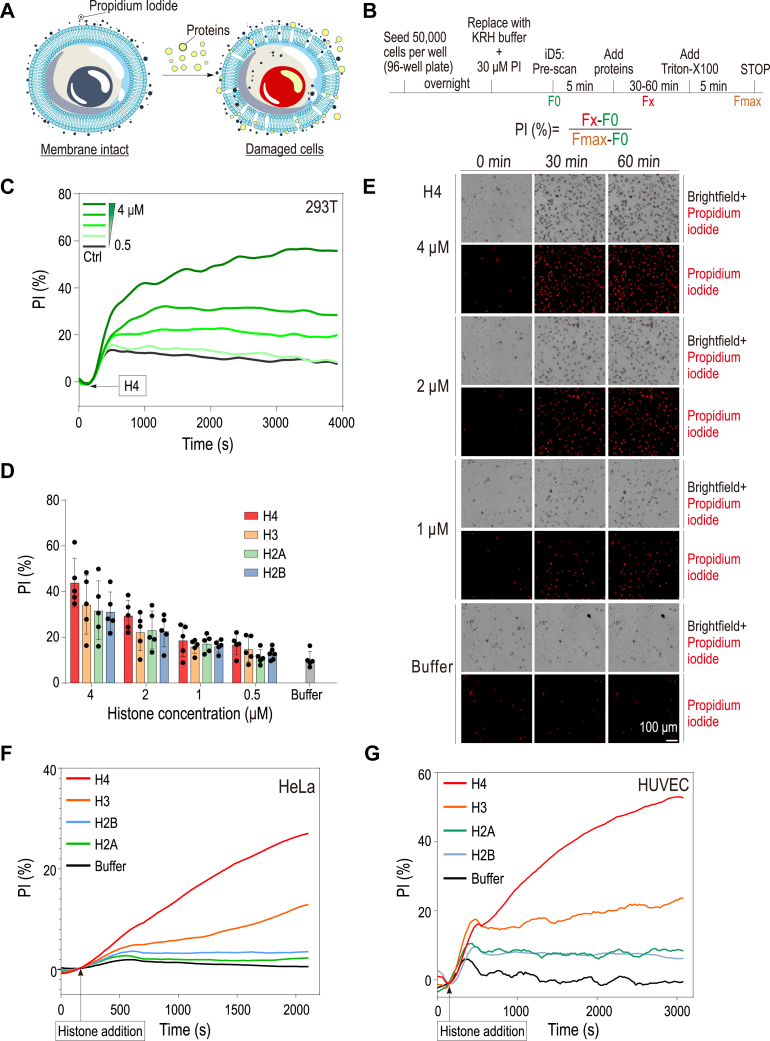
**Extracellular histones exhibit cytotoxicity across various cell types.***A*, schematic of the propidium iodide (PI) exclusion assay, illustrating that only damaged cells are stained by PI, producing detectable signals through fluorescence, indicative of cell membrane compromise. *B*, workflow for the PI exclusion assay, including a detailed protocol and a formula for calculating the percentage of PI-stained cells relative to the maximum staining achieved following detergent treatment, which serves as a control for complete cell lysis. *C*, time-course detection of cytotoxic effects of histone H4 in 293T cells, showing rapid induction of cell death with histone concentrations ranging from 0.5 to 4 μM. *D*, comparative cytotoxicity of different histone types on 293T cells at various concentrations. *E*, PI staining following histone H4 treatment toward HeLa cells, visualizing cell death at various concentrations of histone H4, at two distinct time points. Scar bar: 100 μm. *F*, time-dependent cytotoxicity of histones in HeLa cells, with analysis over a time course at a fixed histone concentration of 2 μM. *G*, cytotoxicity of histones on human umbilical vein endothelial cells (HUVECs) assessed at a histone concentration of 4 μM. The presented results in (*F*) and (*G*) are derived from the integration of triplicate data, resulting in a smoothed curve.

Our unexpected discovery that the globular domains of histones exhibit significant pore-forming capabilities on artificial membranes prompted further investigation into their potential to induce cellular damage in natural cell lines. We utilized a propidium iodide (PI) cytotoxicity assay, leveraging the specific characteristic of PI where it can only penetrate cells and bind to DNA, emitting fluorescence, when the cellular membrane is compromised (Fig. 2*A*). We systematically monitored the progression of fluorescence signal following exposure to histones. To ensure comprehensive cell permeabilization and establish a baseline for maximal PI uptake, Triton X-100 was added at the conclusion of the experiment. This allowed us to accurately quantify the cytotoxic effects of various histones, providing a measure of their potential to disrupt membrane integrity (Fig. 2*B*).

To establish a robust assay system, we initially quantified the cytotoxic effects of histones using the 293T cell line. Our observations indicated that histone H4 significantly compromised cell membrane integrity, resulting in a cell death rate of approximately 60% (Fig. 2*C*). Subsequent comparative analysis of the four core histones revealed consistent cytotoxic effects in 293T cells (Fig. 2*D*). Further investigations were extended to multiple cell lines including HeLa cells, where propidium iodide (PI) staining and cytotoxic assays were conducted post-histone H4 treatment (Fig. 2, *E* and *F*). Our data showed that histone H4 concentrations ranging from 2 to 4 μM induced 60%–80% cell mortality at two distinct time points (Fig. 2*E*). Extended analysis across different cell types, including HeLa and HUVEC cells, confirmed the cytotoxic potential of all core histones (Fig. 2, *F* and *G*).

To elucidate whether the extracellular cytotoxicity of histones differs among various cell types, we evaluated the cytotoxic effects of the four core histones on five distinct cell lines: 293T, HeLa, HUVEC, Vero6, and HepG2 (Fig. S2). Our analysis revealed that all tested histones exerted cytotoxic effects on these cell lines; however, the extent of toxicity was variable. Histone H4 was identified as the most cytotoxic across the majority of cell lines, except for Vero6 cells, where histone H2B demonstrated the highest toxicity (Fig. S2). This variability underscores that histone-mediated cytotoxicity is not uniform across cell types and is not exclusively attributed to any specific histone subtype. Further, we explored the role of positive charge in mediating cytotoxicity by comparing the effects of synthesized positively charged CR20 and negatively charged 3 × FLAG peptides. Despite its positive charge, CR20 exhibited markedly lower cytotoxicity compared to histone H4, with only about 7% cytotoxicity at 2 μM concentration and approximately 20% at 16 μM (Fig. S1*C*). These findings indicate that while positive charge is a contributing factor, it is not the main determinant of cytotoxicity.

### The globular domain of extracellular histones is primarily implicated in inducing lytic cell death

**Figure 3:**
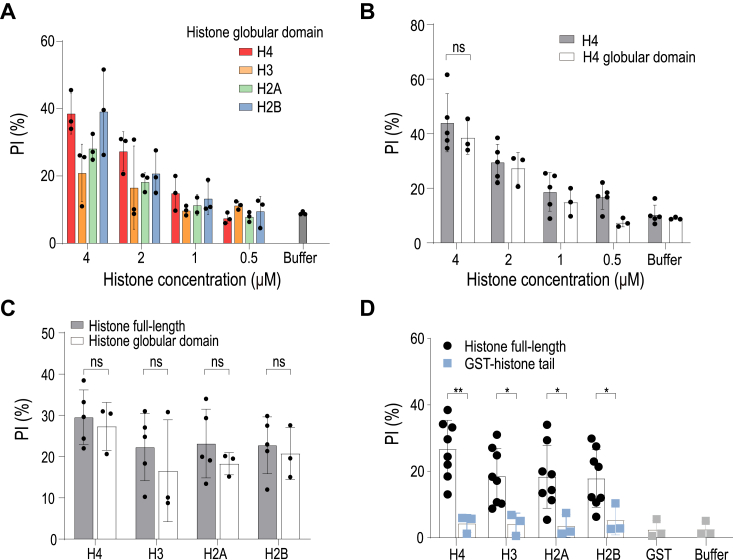
**The globular domain of extracellular histones is primarily responsible for inducing lytic cell death.***A*, cell death induced by the globular domains of four different types of histones in 293T cells. *B*, comparison of the cytotoxicity of full-length histone H4 and its globular domain at varying concentrations. *C*, cytotoxicity comparison between full-length histones and their globular domains, showing similar levels of cytotoxicity for each type. *D*, comparison of cytotoxicity between full-length histones and their tails, revealing that the histone tails exhibit minimal cytotoxicity. *p* values of two-sided Student’s *t* test are shown.

We further analyzed the cytotoxic effects of the extracellular globular domains of all four core histones at various concentrations. The results confirmed that all histones exhibited cytotoxicity in a concentration-dependent manner (Fig. 3*A*). A side-by-side comparison of histone H4 illustrated that the globular domain alone displayed cytotoxicity comparable to that of the full-length protein across different concentrations (Fig. 3*B*). We extended this comparison to all four histones, both full-length and globular domains, and found that the globular domains alone contributed to cytotoxicity identical to that of the full-length proteins, corroborating the results from the liposome leakage assay (Fig. 3*C*). Additionally, we demonstrated that GST-tagged histone tails showed minimal cytotoxicity, consistent with their lack of pore-forming ability (Fig. 3*D*). These findings underscore the crucial role of the globular domain in mediating histone-induced lytic cell death.

### Histone cytotoxicity of individual histones is reduced upon formation of histone H2A/H2B dimers or H3/H4 tetramers

**Figure 4:**
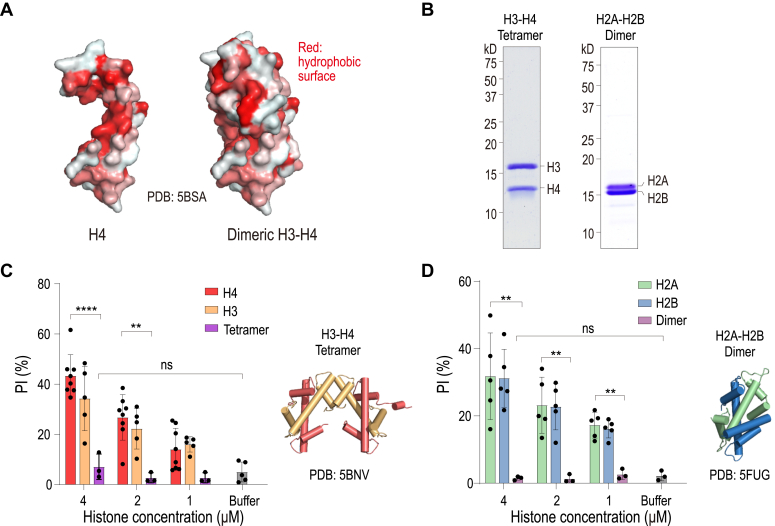
**Histonecytotoxicity of individual histones is reduced upon the formation of histone H2A/H2B dimers or H3/H4 tetramers.***A*, structural representation of H4 and the dimeric H3-H4 complex, shown in surface view with hydrophobic regions highlighted in *red* (PDB: 5BSA (28)). *B*, coomassie *blue* staining of purified H2A-H2B dimers and H3-H4 tetramers. *C*, *Left*: H3-H4 tetramers significantly reduce cytotoxicity compared to H3 and H4 monomers; *right*: structural representation of the H3-H4 tetramer (PDB: 5BNV (29)). *D*, *Left*: H2A-H2B dimers markedly decrease cell death compared to H2A and H2B monomers; *right*: structural representation of the H2A-H2B dimer (PDB: 5FUG (30)). *p* values of two-sided Student’s *t* test are shown.

Soluble individual histones in an aqueous environment tend to adopt misfolded conformations that minimize the exposure of hydrophobic surfaces, which are typically sequestered within the protein core or when the histone is complexed with other histones. Specifically, histone H4, which is relatively small, possesses hydrophobic regions that are generally obscured in its native state upon association with histone H3 (Fig. 4*A*). However, when H4 alone is localized extracellularly, these hydrophobic areas become exposed, which may enhance its potential for interacting with lipid bilayers and promoting membrane disruption. Given our findings of membrane-disrupting and cytotoxic properties of full-length histones and their globular domains, we next sought to investigate whether the H3/H4 tetramer, H2A/H2B dimer native complexes retain any pore-forming capabilities. To this end, we reconstituted recombinant H2A/H2B dimers and H3/H4 tetramers in high salt condition, and they remain in complex form under the physiologically low salt condition (Fig. 4*B*). The cytotoxic assay demonstrated that both the H3/H4 tetramers and the H2A/H2B dimers exhibited significantly reduced cytotoxicity compared to their monomeric or uncomplexed forms (Fig. 4, *C* and *D*). These findings support the hypothesis that the assembly of histone fold pairs that sequester hydrophobic regions effectively diminishes their potential to destabilize lipid membranes.

## Discussion

Beyond their well-known nuclear functions, histones have recently attracted attention for their roles in extracellular physiology and pathology, which are increasingly recognized as significant in various diseases. Previous studies have shown that extracellular histones can induce membrane leakage in certain cell types but not others, although the underlying mechanisms remain elusive. In this study, we aimed to elucidate these mechanisms through a systematic biochemical approach. We employed liposome leakage assays as a model system to evaluate the pore-forming capacities of various histone forms, including individual histone components, their tail regions, and globular domains. Our surprising results indicated that the histone globular domains predominantly contribute to the pore-forming activity. To further investigate the specificity and extent of histone-induced cellular damage, we conducted cytotoxicity assays using combinations of different histone forms. Our findings suggest a model in which the globular domains of extracellular histones play a critical role in disrupting lipid bilayers to mediate cytotoxicity.

One of the key findings from our study is that the globular domains of histones predominantly contribute to membrane disruption. This contrasts with previous studies which highlighted the role of the N-terminal α-helical sequences in histone H4, known for their membrane-anchoring capabilities and subsequent induction of cell death (17). It was shown that histone H4 exhibits high σ scores, indicative of their ability to induce negative Gaussian curvature in membranes, a prerequisite for pore formation as demonstrated by cell-penetrating peptides (17). However, it is well-documented that a histone tail, when fused to a cell-penetrating peptide derived from HIV TAT, can translocate into the nucleus and perform its function without damaging the cell membrane (21). Consistent with these observations, our findings also indicate that the histone tails alone do not display cytotoxic activity (Fig. 3*D*). Furthermore, our results suggest that native pairs of histones, H2A/H2B dimer, and H3/H4 tetramer, serve to shield hydrophobic residues, reducing their cytotoxic potential (Fig. 4). In the context of native chromatin released from neutrophil extracellular traps (NETs), which typically contains soluble tetramers or dimers, this implies that proteolytic events in the extracellular milieu could be crucial for inducing cytotoxicity.

Histone-mediated membrane pore formation is a complex process that may share some mechanistic similarities with other pore-forming proteins (PFPs), although distinct differences are evident due to the unique properties of histones. Common PFPs, such as BAX, BAK, BOK, gasdermins (GSDMs), and MLKL, typically undergo a series of regulated steps involving monomer-to-oligomer transitions, conformational changes, and integration into lipid bilayers to form functional pores (19, 22). These steps are often triggered by interactions with lipids or proteins, proteolytic cleavage, or post-translational modifications such as phosphorylation (19). On the other hand, histones, being highly basic proteins, may initiate membrane interactions through their positively charged regions, which attract the negatively charged phosphate groups of lipid molecules on the cell membrane (17). This electrostatic interaction may be the first critical step toward histone-mediated membrane disruption. Following this initial contact, the hydrophobic regions within the histone globular domain regions may engage with the lipid acyl chains, facilitating further penetration into the bilayer. This interaction disrupts the structural integrity of the membrane, potentially leading to the formation of transmembrane channels. Atomic Force Microscopy (AFM) and other advanced imaging techniques have shown that upon interaction with membranes, histones cause visible deformations such as dents and disruptions (17). These deformations are indicative of the mechanical stress exerted by histones on the lipid bilayer, which may lead to pore formation or extensive membrane destabilization. Such visual evidence supports the hypothesis that histones can induce membrane pores, albeit possibly through mechanisms that differ from classical PFPs due to the intrinsic properties of histones and their modes of interaction with lipid membranes.

Our study reveals that different histones induce varying degrees of toxicity across cell types. The mechanisms by which certain cells circumvent the cytotoxic effects of extracellular histones are contingent upon factors such as lipid composition, and the localization and conformation of transmembrane proteins (23). Specifically, lipid composition may influence histone penetration, either directly, or indirectly by recruiting different lipid-binding proteins that can accommodate various transmembrane protein functions or by attracting proteins that sense membrane curvature or packing defects. Alternatively, cells may engage the ESCRT-dependent membrane repair mechanism to mitigate histone-induced damage (23). This pathway not only promotes membrane repair but also initiates downstream immune responses. In a comparable manner, the cytotoxic impacts of MLKL and gasdermins are regulated through their interactions with the ESCRT-III complex (23). The ESCRT complex plays a crucial role in the removal of these pore-forming proteins such as histones by facilitating their exocytosis through vesicular transport, thereby modulating cellular susceptibility to pore-forming protein-induced cytotoxicity (19).

In conclusion, our novel identification of the globular domain of extracellular histones as a primary agent of membrane disruption warrants further detailed structural analysis. This insight opens avenues for the investigation of histone-binding inhibitors as potential therapeutic agents. By advancing our comprehension of these mechanisms, we enhance our understanding of the pathological roles that histones play in conditions like sepsis and inflammation, thereby facilitating the development of targeted interventions to ameliorate their detrimental effects.

## Experimental procedures

### Cells and cell culture

Human cells 293T, HeLa, HUVEC, Vero6, and HepG2 were cultured in DMEM supplemented with 10% FBS and 2% Penicillin/Streptomycin. Cells were maintained in an incubator at 37 °C with 5% CO_2_. The HUVEC cell line was purchased from the China Infrastructure of Cell Line Resource, Vero6 was a gift from Dr Yingli Wu's lab, and 293T, HeLa, and HepG2 were obtained from Dr Xuxu Sun's lab. There were no signs of *mycoplasma* contamination in cell lines.

### Purification of histones and the globular domain of histones

Human core histones and *Xenopus* globular domains were overexpressed in *Escherichia coli* strain BL21 (DE3) using a 2 × YT medium. Expression was induced by adding 0.2 mM isopropyl β-D-1-thiogalactopyranoside (IPTG) once the optical density at 600 nm reached 0.6, and the culture was incubated for an additional 4 h at 37 °C. Post-expression, cells were harvested, and the cell pellets were resuspended in a wash buffer consisting of 50 mM Tris-Cl (pH 7.5), 100 mM NaCl, 1 mM EDTA, 1 mM benzamidine.HCl, 5 mM β-mercaptoethanol (β-ME), and 0.4 mM PMSF. Cell lysis was achieved using a high-pressure homogenizer (Union UH-06). Subsequently, the cell pellets were washed with the same buffer supplemented with 1% Triton X-100 to remove membrane components. The histones were first resuspended in DMSO and then solubilized in an unfolding buffer composed of 7 M Guanidinium.HCl, 20 mM Tris-Cl (pH 7.5), and 10 mM DTT. The resulting extract was dialyzed against SAU 200 buffer, which includes 7 M urea, 20 mM sodium acetate (pH 5.2), 200 mM NaCl, 1 mM EDTA, and 5 mM β-ME, to reduce the chaotropic agent concentration gently and prepare the proteins for chromatographic purification. Following dialysis, the proteins were applied to a Hi-Trap SP-Sepharose column (GE Healthcare) under denaturing conditions. Protein fractions eluted from the column were analyzed using 14% SDS-PAGE and fractions showing the desired protein bands were pooled and dialyzed against a buffer containing 5 mM β-ME. The purity and concentration of the final purified histones were assessed by running comparative SDS-PAGE analyses. The gels were stained by Coomassie Blue and scanned with an EPSON V850 Pro scanner or Bio-Rad Image Lab system (6.1.0 build 7).

### Reconstitution of histone dimers and tetramers

The process for reconstituting histone dimers begins by lyophilizing equimolar amounts of histones H2A and H2B, which are then solubilized and mixed in an unfolding buffer. The mixture is subsequently dialyzed into a refolding buffer containing 2 M NaCl, 10 mM Tris-Cl (pH 7.5), 1 mM EDTA, and 5 mM β-ME overnight. After being concentrated and with aggregates removed, the sample is loaded onto a Superdex 200 column (GE Healthcare). The elution fractions are analyzed using 14% SDS-PAGE to identify and pool the desired fractions, which are then concentrated further. For the reconstitution of histone tetramers, histones H3 and H4 are similarly processed.

### Cloning, expression, and purification of histone tails

The cDNA sequences encoding the tail regions of human histones were successfully cloned into the pRET-GST expression vector between XhoI and NotI restriction sites. The design of the vector also includes an upstream-engineered TEV protease cleavage site, situated at the N-terminus of the expressed fusion protein, to enable subsequent proteolytic release of the histone tails from the GST tag. Details of the cloning procedure and vector map are provided in Table S1.

GST fused histone tails were individually expressed in BL21CodonPlus-RIL cells and purified as described. Briefly, cells were cultured in 2 L of LB media at 37 °C until OD_600_ reached 0.4 to 0.6. Protein expression was induced by the addition of 0.2 mM IPTG, and cells were cultured at 16 °C overnight. Cells were lysed in 2 M GST buffer containing 2 M NaCl, 10 mM Tris.Cl pH7.5, 20 mM Na_3_PO4 pH 6.8, 0.01% NP40, and 1 mM β-ME, then sonicated and centrifuged at 14,000 rpm at 4 °C for 10 min. The supernatant was incubated with glutathione agarose beads (Shanghai Chuzhi Bio, cat. #SA008010) in 1M GST buffer (2 M NaCl, 10 mM Tris.Cl pH7.5, 20 mM Na_3_PO_4_ pH6.8, 0.01% NP40 and 1 mM β-ME) for 4 h at 4 °C on a SRT roller. Protein was eluted in a GST elution buffer containing 25 mM Tris.Cl pH8.0, 50 mM NaCl, 10 mM β-ME and 10% glycerol supplemented with 100 mM Glutathione. After elution, proteins are dialyzed back into the same GST elution buffer. Finally, the purified proteins are concentrated, flash-frozen in liquid nitrogen, and stored at −80 °C.

### Liposome preparation

Liposome prepared as previously described (20, 24). The lipids used in this study were phosphatidylcholine (POPC) (Avanti, cat. #50457) and phosphatidylethanolamine (POPE) (Avanti, cat. #850757). Briefly, the lipid mixtures POPC: POPE (molar ratio 80:20) were solubilized in chloroform and then dried to form a lipid film on the wall of a glass tube. The dried lipid film was resuspended in RB150G10 containing 25 mM HEPES (pH 7.5), 150 mM KCl, 10% glycerol, supplemented with 2% nonionic surfactant octyl glucoside (OG) (Goldbio, cat. #O-110–50), 0.5 mM TCEP, and 50 mM sulforhodamine B (Invitrogen, cat. #S1307). The suspension was vortexed for 5 min to completely mix the lipids in the buffer. The resulting lipid suspensions were submitted to PD MiniTrap G25 to form dye-containing liposomes by removing OG and free sulforhodamine B. After forming dye-containing liposomes, the size of the liposomes was measured using dynamic light scattering (DLS) on a Zetasizer Nano ZS (Malvern Instruments).

### Liposome leakage assay

In the liposome leakage assay, liposomes encapsulating self-quenched sulforhodamine B molecules were utilized to monitor leakage events. The principle of this assay is based on the detection of increased fluorescence emission at 570 nm from sulforhodamine B when excited at 530 nm. This increase in fluorescence occurs as the dye, initially in a self-quenched state due to high concentration inside the liposomes, gets diluted upon leakage into the surrounding medium.

The liposome leakage assay was performed as previously described with minor modifications (20, 25). The assay was conducted using a SpectraMax iD5 multi-mode microplate reader from Molecular Devices, with measurements taken in a 96-well black/clear flat bottom microplate (Corning, cat. # 3631). The excitation and emission wavelengths utilized for the assay were set at 544 nm and 590 nm, respectively. Sulforhodamine B-labeled liposomes were prepared at a final lipid concentration of 250 μM. The fluorescence baseline of sulforhodamine B was established through a pre-scan over 5 min with readings taken every 28 s. Following this, fluorescence was monitored for a period ranging from 30 to 60 min post the addition of proteins, indicative of any interaction or disruption caused by the proteins to the liposome structure. All experimental measurements were carried out at a controlled temperature of 37 °C. Fluorescence emissions were recorded at each time point and denoted as Fx. For reference, the fluorescence emission of untreated liposomes was recorded as F0, and the emission from liposomes completely solubilized with 2% Triton X-100 was taken as Fmax. To quantify the percentage of leakage from the liposomes, the following formula was used: dye release (% of max)= (F_x_-F_0_) × 100/(F_max_-F_0_). The obtained dye-releasing curves were smoothed using a technique that incorporated 4 to 10 neighboring data points on each side to enhance data representation.

### Propidium iodide exclusion assay

The propidium iodide exclusion assay was performed as previously described (26). Briefly, this assay utilized a Krebs-Ringer-HEPES (KRH) buffer composition consisting of 25 mM HEPES at pH 7.5, 112.6 mM NaCl, 5 mM KCl, 1 mM KH_2_PO_4_, 1 mM MgCl_2_, 1.2 mM Na_2_SO_4_, and 2 mM CaCl_2_, supplemented with 30 μM propidium iodide (Sigma, cat. #P4170). Propidium iodide alone has an excitation peak near 500 nm and an emission peak near 625 nm. Upon binding to DNA, there is a notable redshift, with excitation and emission peaks moving to 540 nm and 640 nm, respectively.

The experiments were carried out using a SpectraMax iD5 multi-mode microplate reader (Molecular Devices) equipped with a 96-well microplate from Corning Inc. The chosen wavelengths for excitation and emission during the measurements were 500 nm and 625 nm, respectively. For the cytotoxicity assessment, cells were seeded in 96-well microtiter plates at a density of 50,000 cells per well, 24 h or at least 8 h prior to experimentation.

Prior to the introduction of test proteins, the cell culture medium was replaced with 80 μl of KRH buffer containing 30 μM propidium iodide. An initial fluorescence scan was performed for 5 min at 28-s to 37-s intervals to establish the baseline fluorescence (F0). Subsequently, varying concentrations of test proteins or control buffers were administered using a multichannel pipette. Fluorescence readings were taken every 28 s for a duration of 30 to 60 min post protein addition to monitor changes (Fx). During these intervals, the plate was maintained in a 37 °C air incubator.

An increase in fluorescence intensity indicates a compromise in cell membrane integrity, indicative of cytotoxic effects. The maximal change in fluorescence was equated to 100% cell death. At the conclusion of the experiment, 2% Triton X-100 was added to each well to permeabilize all cells and ensure complete DNA labeling with propidium iodide. A final 10-min fluorescence measurement was taken to establish the fluorescence value corresponding to 100% cell death (Fmax).

Percent cytotoxicity was calculated using the formula: PI (%) = (F_x_-F_0_) × 100/(F_max_-F_0_), where F0 is the initial fluorescence, Fmax is the fluorescence post-Triton X-100 addition, and Fx represents the fluorescence at any given time point during the assay. The obtained cytotoxicity curves were smoothed using a technique that incorporated 4 to 10 neighboring data points on each side to enhance data representation.

### Propidium iodide staining assay

The procedure of propidium iodide staining assay was almost performed as propidium iodide cytotoxicity assay. Briefly, cells were cultured in 96-well microtiter plates (50,000 cells per well) the day before and replaced the medium with KRH buffer containing 30 μM propidium iodide. After treated with proteins or buffer, the brightfield and PI were both recorded by ImageXpress Pico (Molecular Devices) under 10 × microscope. All measurements were performed at 37 °C.

### Calculation of isoelectric point and net charge

The isoelectric point and net charge of histone at pH 7.4 were calculated using the Prot pi online tool (https://www.protpi.ch/).

### Quantification and statistical analysis

These statistical analyses were performed using the GraphPad Prism 8 software. Data are presented as mean ± SD. Statistical significance was determined using the following criteria: ∗*p* < 0.05, ∗∗*p* < 0.01, ∗∗∗*p* < 0.001, ∗∗∗∗*p* < 0.0001.

## Data availability

The main data supporting the findings of this study are available within the article and in Supporting information. Published datasets from the Protein Data Bank used in this study: 5BSA, 5BNV and 5FUG. All plasmids and materials generated in this study are available from the lead contact with a completed Materials Transfer Agreement.

## Supporting information

This article contains supporting information (27).

## Conflict of interest

The authors declare that they have no conflicts of interest with the contents of this article.
