# Supplementary material for: The globular domain of extracellular histones mediates cytotoxicity via membrane disruption mechanism
Source: J Biol Chem. 2024 Nov 28;301(1):108038. doi: 10.1016/j.jbc.2024.108038 (PMC11732447; doi:10.1016/j.jbc.2024.108038)
Supplement: Supporting information [file mmc1.docx]

**SUPPORTING INFORMATION**

**The globular domain of extracellular histones mediates cytotoxicity *via* membrane disruption mechanism**

YixuanPan^1,‡^, MengyuanPeng^1,‡^, MindanTong^2^, YueHe^1^, MinHao^1^, He LilianGao^1^, YiminLao^1^, JingdongXue^1^, MeiyangLiu^1^, QingZhong^2^, XiaoxiaLiu^2,∗^, and BingLi^1, ∗^

**SUPPLEMENTAL INVENTORY**

**SUPPLEMENTAL FIGURES**

Figure S1. Electrostatic interactions contribute to histone-induced lytic cell death.

Figure S2. Extracellular histones show differential cytotoxicity depending on cell type.

**SUPPLEMENTAL TABLE**

Table S1 List of plasmids used in this study

**SUPPLEMENTAL REFERENCE**

**SUPPLEMENTAL FIGURES**

**Figure S1. Electrostatic interactions contribute to histone-induced lytic cell death.**

(A) Isoelectric point and net charge at pH 7.4 for full-length histones, the tail region, and the globular domain.

(B) Coomassie blue staining of full-length histones, the globular domain and the tail region that purified from *E.coli*.

(C) Cytotoxicity of CR20 and 3×FLAG peptides towards 293T cells, assessed using a propidium iodide cytotoxicity assay. CR20 consists of one cysteine (C) and twenty arginines (R), while 3×FLAG is composed of the sequence DYKDDDDKGDYKDDDDKGDYKDDDDK.

**Figure S2. Extracellular histones show differential cytotoxicity depending on cell type.**

(A) Time-course analysis of extracellular histone cytotoxicity on two representative cell types, with histone concentrations ranging from 1 to 8 µM.

(B) Heatmap displaying the cytotoxicity of four different histone types across five cell lines, with histone concentration set at 2 µM.

**SUPPLEMENTAL TABLE**

**Table S1 List of plasmids used in this study**

| Name | Backbone | Plasmids Description | Source |
| --- | --- | --- | --- |
| pBL113 | pET-21a | pET-21a human H3.3B | Parvin JD |
| pBL114 | pET-21a | pET-21a human H2A | Parvin JD |
| pBL115 | pET-21a | pET-21a human H2B | Parvin JD |
| pBL116 | pET-21a | pET-21a human H4 | Parvin JD |
| pBL455 | pET | pET-*Xenopus* gH3 (27-135) | Richmond |
| pBL456 | pET | pET-*Xenopus* gH4 (20-102) | Richmond |
| pBL457 | pET | pET-*Xenopus* gH2A (13-118) | Richmond |
| pBL458 | pET | pET-*Xenopus* gH2B (22-122) | Richmond |
| pYX035 | pRET | pRET-GST-2×FLAG | [^27^](#_ENREF_1) |
| pYX143 | pYX035 | pRET-GST-human H2A N (1-17) | this study |
| pYX144 | pYX035 | pRET-GST-human H2B N (1-31) | this study |
| pYX145 | pYX035 | pRET-GST-human H3 N (1-45) | this study |
| pYX146 | pYX035 | pRET-GST-human H4 N (1-37) | this study |

**SUPPLEMENTAL REFERENCE**

27. Xue J, Lv S, Yu M, Pan Y, Li N, Xu X*, et al.* ARID1A IDR Targets EWS-FLI1 Condensates and Finetunes Chromatin Remodeling. *Protein Cell* , 2024, pwae029
