# Supplementary material for: The globular domain of extracellular histones mediates cytotoxicity via membrane disruption mechanism
Source: J Biol Chem. 2024 Nov 28;301(1):108038. doi: 10.1016/j.jbc.2024.108038 (PMC11732447; doi:10.1016/j.jbc.2024.108038)

Figure S1

A

Histone	Isoelectric point	Net charge at pH 7.4
H2A	11.226	+16.407
H2B	10.155	+17.327
H3.3	11.650	+19.198
H4	11.762	+17.315
H2A tail	12.574	+6.208
H2B tail	10.591	+11.855
H3.3 tail	12.804	+13.613
H4 tail	12.507	+8.249
gH2A	10.325	+9.372
gH2B	9.702	+5.325
gH3.3	9.934	+5.435
gH4	10.658	+8.918
CR20 peptide	13.432	+19.639
3xFLAG peptide	4.131	- 7.262

B

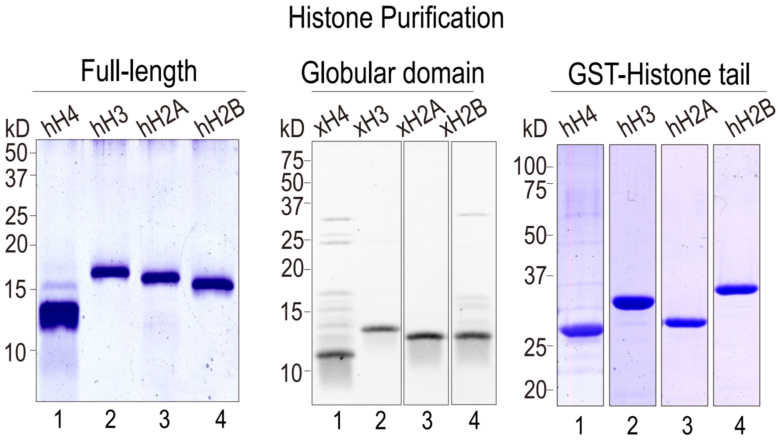

C

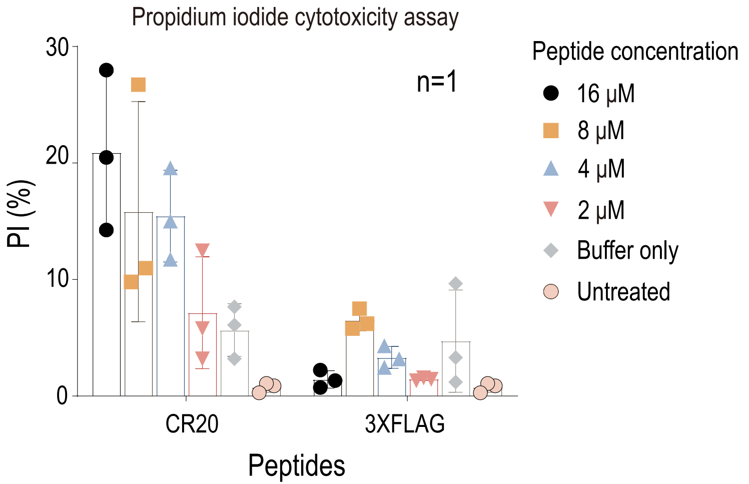

Supplement: Supplementary Figure 1 [file mmc2.pdf]
