# Supplementary figures and images for: The globular domain of extracellular histones mediates cytotoxicity via membrane disruption mechanism
Source: J Biol Chem. 2024 Nov 28;301(1):108038. doi: 10.1016/j.jbc.2024.108038 (PMC11732447; doi:10.1016/j.jbc.2024.108038)

**Figure S2****A**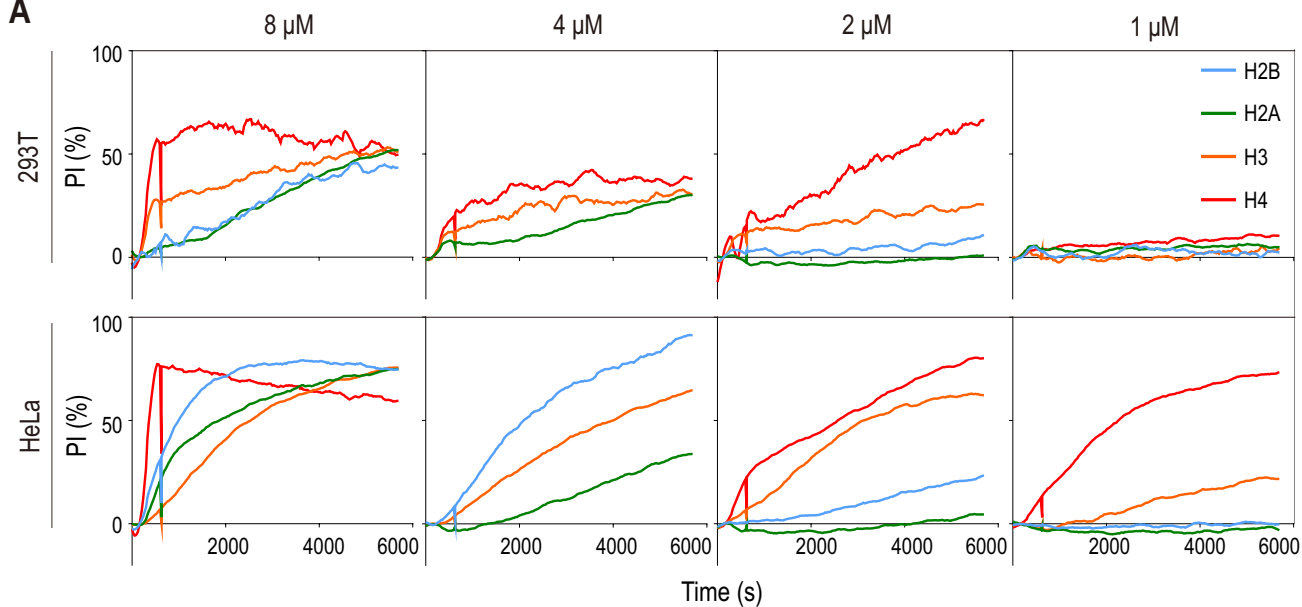**B**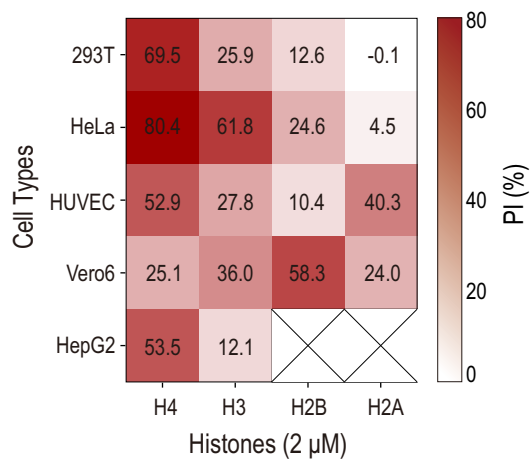

Supplement: Supplementary Figure 2 [file mmc3.pdf]
